# Peroxiredoxin 6: The Protector of Male Fertility

**DOI:** 10.3390/antiox7120173

**Published:** 2018-11-24

**Authors:** Cristian O’Flaherty

**Affiliations:** Departments of Surgery (Urology Division) and Pharmacology and Therapeutics, Faculty of Medicine, McGill University and the Research Institute, McGill University Health Centre, Montréal, QC H4A 3J1, Canada; cristian.oflaherty@mcgill.ca

**Keywords:** spermatozoa, oxidative stress, reactive oxygen species, fertilization, sperm capacitation

## Abstract

The spermatozoon is a terminal cell with the unique purpose of delivering the paternal genome to the oocyte during fertilization. Once spermatozoa enter into the female reproductive tract, they count on only the antioxidant protection that they received during spermatogenesis and epididymal maturation. Peroxiredoxins (PRDXs), particularly PRDX6, are important players in the antioxidant protection and regulation of reactive oxygen species (ROS) levels in spermatozoa. PRDX6, through its peroxidase and calcium-independent phospholipase A_2_ activities, plays a major role in the regulation of ROS to maintain viability and motility and allow the spermatozoon to achieve fertilizing ability during the complex process of capacitation. The absence of PRDX6 is sufficient to promote abnormal reproductive outcomes in mice that resemble what we observe in infertile men. Indeed, Prdx6^−/−^ spermatozoa display low motility and severe DNA damage, which is translated into reduced ability to fertilize oocytes in vitro or produce a low number of pups compared to wild-type controls. This review focuses on the role of PRDX6 as the primary antioxidant enzyme that protects the spermatozoon from oxidative-stress-associated damages to protect the paternal genome and assure fertility.

## 1. Introduction

The mammalian spermatozoon is a specialized cell with only one purpose: fertilize and deliver the paternal genome to the ovulated oocyte. To accomplish these tasks, the spermatozoon needs to be able to move and to achieve fertilizing capacity to recognize the oocyte. These essential activities for the survival of any species are susceptible to failure. Infertility is a significant human health problem that affects 1 in 6 couples worldwide, and the infertile population has been increasing over the past few decades [1]. The underlying cause in half of these cases can be traced to men [1,2]. 

Spermatozoa are very sensitive to high levels of reactive oxygen species (ROS), which promote lipid peroxidation [3]. This peroxidative damage is mainly due to the high levels of polyunsaturated fatty acids of their plasma membrane [3,4]. Moreover, the enzymatic antioxidant protection in human spermatozoa is limited. There is relatively little cytoplasm containing superoxide dismutase 1 (SOD1). They do not have catalase [5,6], glutathione peroxidase (GPX) 1, GPX2, GPX3, and GPX5 [7,8], and the mitochondrial mGPX4 is enzymatically inactive in mature spermatozoa [9,10]. Mitochondrial GPX4 is essential during the spermatogenesis to produce normal spermatozoa. This enzyme is needed to build the mitochondrial sheath, an envelope that surrounds the mitochondrial helix in the flagellum, and its absence is associated with male infertility [10,11]. 

Then, how do healthy spermatozoa control ROS levels and avoid oxidative damage? Spermatozoa contain SOD2, nuclear GPX4, thioredoxin (TRX), TRX reductase (TRD), and the specific sperm TRX1 and 2 [6,12,13]. We found that the six members of the peroxiredoxins (PRDX) family are localized in different sperm compartments (head, mitochondrial sheath, and flagellum), where TRX and TRD (enzymes needed for reactivation of PRDX activity) are also found. PRDXs react with different hydrogen peroxides, organic peroxides, and peroxynitrite in spermatozoa [6,12,13]. Moreover, we recently reported that reduced levels of PRDXs are associated with male infertility [14]. Thus, PRDXs play an important role in the protection of spermatozoa against oxidative stress. Notably, PRDX6 stands alone as the major player in the protection of spermatozoa against oxidative stress. Indeed, we observed that spermatozoa from infertile patients with either clinical varicocele or idiopathic infertility had lower levels of PRDX6, which correlated with low motility and high levels of lipid peroxidation and DNA damage compared to fertile controls [14]. Animals studies indicated that male mice lacking Prdx6^−/−^ produced a lower number of pups than the wild-type controls [15]. PRDX6 is a moonlighting enzyme with peroxidase and calcium-independent phospholipase A_2_ (iPLA_2_) activities [16,17]. The peroxidase activity is necessary to remove hydrogen peroxide (H_2_O_2_), other hydroperoxides, and peroxynitrite (ONOO^−^), and the iPLA_2_ activity is essential to repair oxidized membranes [16]. Indeed, PRDX6 iPLA_2_ removes the peroxidized phospholipid, and the lysophosphatidylcholine acyl transferase activity (also present in PRDX6) replace it with a nonoxidized phospholipid [17].

## 2. PRDX6 and Male Infertility

Infertility is on the rise, with couples that struggle with infertility increasing from 8% in the 1980s to almost 17% worldwide [1,2]. The prevalence of idiopathic infertility or that associated with clinical varicocele (a dilation of pampiniform plexus veins) is 23% and 43%, respectively [18]. These are the most common causes of male infertility. We observed that these patients had reduced amounts of PRDXs in seminal plasma and spermatozoa compared to fertile donors [14]. Notably, thiol-oxidized (an inactive) PRDXs levels and the presence of high molecular mass complexes (containing the inactive PRDX6-SO_2_) [12] were also elevated in sperm from infertile men [14]. Due to the low total amount and the oxidation status of PRDXs, very little antioxidant protection (less than 20%) remains, which explains the impairment of sperm function and poor DNA quality observed in these patients [14]. PRDX6 calcium-independent phospholipase A_2_ (iPLA_2_) and peroxidase activities are necessary to maintain sperm viability and integrity of the paternal genome [19]. The integrity of the paternal genome is essential to assure fertility. 

**Figure 1:**
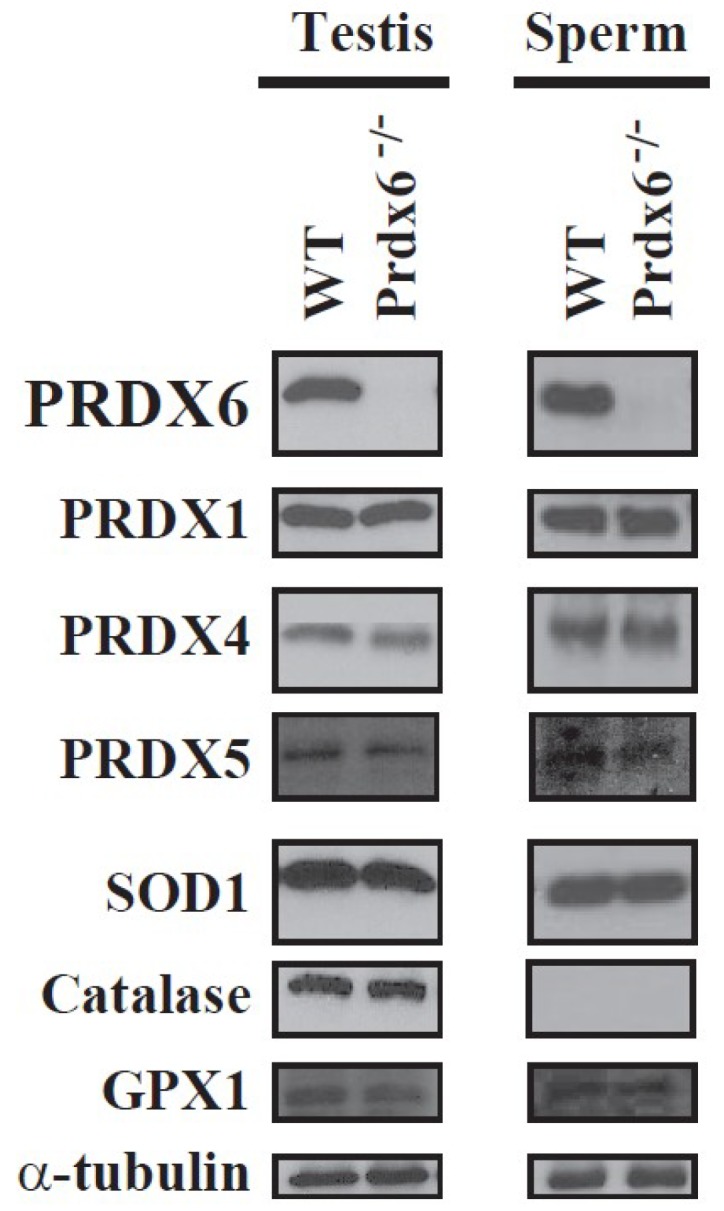
Expression of antioxidant enzymes in wild-type and Prdx6^−/−^ testis and spermatozoa. No compensatory mechanisms by overexpression of other antioxidant enzymes were observed due to the absence of peroxiredoxin 6 (PRDX6) in testes or spermatozoa. Sodium dodecyl sulfate polyacrylamide gel electrophoresis (SDS-PAGE) and immunoblotting of different antioxidant enzymes in C57Bl6J wild-type (WT) or Prdx6^−/−^ testis and spermatozoa were done as before [12,15]. α-tubulin was used as loading control (*n* = 4). SOD1: superoxide dismutase 1; GPX1: glutathione peroxidase 1.

Spermatozoa from Prdx6^−/−^ mice are sensitive to in vitro and in vivo oxidative stress, showing higher levels of lipid peroxidation, S-glutathionylated and oxidized (and non-functional) proteins, abnormal sperm chromatin structure, and a sperm phenotype similar to that of infertile men (abnormal reproductive outcomes, low motility, and oxidative damage in proteins and DNA) [15]. We observed that Prdx6^−/−^ spermatozoa and testes did not have higher levels of other antioxidant enzymes than wild-type (WT) mice (Figure 1). Thus, no compensatory antioxidant enzymes were upregulated in the absence of PRDX6 [15]. 

Excess of reduced glutathione (GSH) is a cause of reductive stress, which results in an imbalance of the redox status and cytotoxicity [20]. Because spermatozoa contain very little amounts of GSH, this tripeptide is rapidly consumed in cases of oxidative stress, such as the one occurring in the Prdx6^−/−^ spermatozoa. Indeed, the high levels of S-glutathionylation observed in Prdx6^−/−^ spermatozoa accounts for this rapid depletion of GSH [15].

Interestingly, we found that aging worsens the Prdx6^−/−^ phenotype, with a severe reduction in fertility and greater damage to sperm chromatin compared to age-matched WT controls [21]. These findings are worrisome as some men plan to have children in their 40s or beyond due to professional or work priorities. Increasing evidence shows that aging men have increased numbers of abnormal spermatozoa [22]. Thus, this deliberate delay may negatively impact fertility as sperm quality decreases as men age [23,24,25].

Recently, it was reported that a 50% reduction in fertilization rate was noted when mouse spermatozoa were treated with conoidin A, a specific inhibitor of 2-Cys PRDXs, compared to untreated controls [26]. Our in vitro studies demonstrated that fertilization rates were reduced by 80% when using Prdx6^−/−^ compared to WT spermatozoa, and Prdx6^−/−^ spermatozoa were unable to produce blastocysts in vitro [27]. Thus, these results indicate that one of the causes of in vitro fertilization failure might also be associated with activity dysfunction of PRDXs, particularly PRDX6, in spermatozoa.

The spermatozoa are unable to move or even fertilize an oocyte when they leave the testis. They must undergo a series of biochemical and morphological transformations to sustain these sperm functions. This process is called sperm maturation and it occurs in the epididymis, an organ composed of a 3–6-meter-long complex and convoluted tubule in humans [28]. During the epididymal transit, the removal of part of the cytoplasm makes the spermatozoa more vulnerable to oxidative stress because it leads to SOD and other cytosolic antioxidant enzymes remaining in low amounts in the maturing spermatozoon [29]. We observed that adult male Sprague–Dawley rats exposed to an in vivo oxidative stress generated by tert-butyl hydroperoxide (tert-BHP) had spermatozoa with decreased motility and increased levels of DNA oxidation and lipid peroxidation along with increased amounts of PRDX1 and PRDX6 compared to control rats [30]. Interestingly, the amount of SOD did not change due to this in vivo treatment. Sperm PRDXs were highly oxidized and therefore inactivated. There was a differential regulation in the expression of PRDX1 and PRDX6 in the epididymis, which suggests a segment-specific role of PRDXs to fight against oxidative stress [30]. These findings are interesting in light of the fact that the spermatozoon is transcriptionally silent; thus, an increase in the protein content should be due to the transfer of proteins from the epididymal epithelium. This increase in the PRDX content in the maturing spermatozoa is a consequence of the response of the epididymis to the in vivo oxidative stress to protect the spermatozoa during their transit through this organ. The epididymis produces epididymosomes, vesicles of different sizes containing proteins with a diverse function from cell adhesion, energy metabolism, and antioxidant protection [31,32]. Through epididymosomes, the epididymis delivers the proteins needed by the maturing spermatozoa depending on the stage of maturation and in response to the environmental conditions. Indeed, the fact that PRDX1 and PRDX6—but not SOD—were increased in sperm from the tert-BHP-treated rats support the specific delivery actions of epididymosomes depending on the type of oxidative stress present in the epididymis [30].

## 3. PRDX6 Peroxidase and Phospholipase A_2_ Activities are Important for Sperm Quality

The spermatozoon is a terminal cell with the unique purpose of delivering the paternal genome to the oocyte during fertilization. They need to survive in the female reproductive tract, move to colonize the oviduct to be capacitated and then to find the oocyte, penetrate the zona pellucida, fuse with the oolemma, and finally form the male pronucleus just before the syngamy. Once the sperm enters into the female genital tract, they leave behind the antioxidant protection that the seminal plasma provides. Then, it is up to the internal antioxidant enzymes the spermatozoon carries to protect the cell during this journey in the female reproductive tract until fertilization occurs. 

Sperm motility is severely affected by oxidative stress. The machinery that makes spermatozoon to move is the target of ROS and lipid peroxidation. Enzymes that produce energy and tubulin, the structural protein in the sperm flagellum, are directly oxidized by ROS or modified by ROS-dependent protein modifications and lipid peroxidation. Prdx6^−/−^ spermatozoa are less viable with lower motility than the WT controls [27]. They have high levels of carbonyl groups and S-glutathionylation than WT controls, indicating the oxidative stress that is going on in the cell, and damage sperm proteins [15]. The significant lower motility observed in Prdx6^−/−^ spermatozoa compared to WT controls can be due to the inhibition of enzymes required to generate energy and the thiol oxidation of tubulin [33,34,35]. Unsaturated fatty acids are highly susceptible to peroxidation, and lipid peroxidation has been associated with impairment of sperm motility and infertility [3,36].

We observed a lower percentage of WT oocyte with pronuclear formation produced when inseminated with Prdx6^−/−^ compared to WT spermatozoa during in vitro fertilization [27]. Notably, there was a delay in the formation of pronuclei as a similar number of oocytes with male and female pronuclei were observed at 6 h or 8–10 h after in vitro fertilization using WT or Prdx6^−/−^ spermatozoa, respectively. This finding indicates damage to the paternal genome. Indeed, Prdx6^−/−^ spermatozoa have abnormal chromatin structure, displaying significant higher DNA damage (oxidation and fragmentation) and low levels of protamines (small basic proteins that replaced histones during spermiogenesis to make the sperm nucleus smaller), which result in lower DNA compaction compared to WT controls [15]. This damage of the paternal genome is responsible for the smaller number of pups generated by natural mating with Prdx6^−/−^ males and WT females [15] or the incapacity to produce healthy embryos in vitro. Certainly, although we obtained a reduced number of zygotes and 2-cell embryos using Prdx6^−/−^ spermatozoa and WT oocytes, these embryos did not develop to blastocysts (preimplantation embryos) [27]. This phenomenon has been observed when using spermatozoa exposed to radiation [37,38].

Our studies suggest that the PRDX6 iPLA_2_ activity is a major factor in the protection of the sperm DNA. We also found lower fertilization rates and percentage of blastocysts when we pretreated WT spermatozoa with 1-hexadecyl-3-(trifluoroethyl)-*sn*-glycero-2-phosphomethanol lithium (MJ33) [27]. The sperm DNA oxidation levels were higher in human spermatozoa treated with MJ33 compared to nontreated controls or even in spermatozoa treated with conoidin A, an inhibitor of 2-Cys PRDXs, or with ezatiostat, an inhibitor of glutathione-S-transferase Pi, which is an enzyme that participates in the reactivation of the PRDX6 peroxidase activity [19]. Thus, it is possible that failure to achieve fatherhood resides in the inactivation of PRDX6 iPLA_2_ in spermatozoa of infertile men.

The origin of the oxidative damage responsible for the reduction of viability, motility, and increased DNA damage is associated with an increase in the production of mitochondrial superoxide anion due to mitochondrial dysfunction in human spermatozoa. The inhibition of both PRDX6 iPLA_2_ and peroxidase activities resulted in higher mitochondrial superoxide production compared to those obtained when 2-Cys PRDXs were inhibited [19]. The production of 4-hydroxy-2,3-nonenal (4-HNE) was significantly higher in spermatozoa treated with MJ33 or ezatiostat compared to conoidin A, the inhibitor of the 2-Cys PRDXs. These findings indicate that both PRDX6 peroxidase and iPLA_2_ activities are essential to control ROS levels and avoid damage associated with lipid peroxidation in the spermatozoa. 4-HNE is a lipid peroxidation product with mutagenic properties that reacts with all four DNA bases, with guanosine the most affected [39]. Thus, ROS promote DNA mutations by directly oxidizing DNA bases (8-hydroxy-2′-deoxyguanosine) or by generating 4-HNE that will form DNA adducts [39,40] responsible for male infertility.

## 4. PRDX6 and Sperm Capacitation

Ejaculated spermatozoa are unable to fertilize oocytes. They must reside for several hours in the oviduct of the female genital tract to acquire fertilizing ability. Low levels of ROS are necessary for the spermatozoon to acquire fertilizing ability [41,42]. They trigger and regulate phosphorylation events during capacitation to allow the spermatozoon to undergo the acrosome reaction and fertilize the oocyte [42,43,44]. Sperm capacitation requires a tightly controlled redox signaling [42,45], which is disrupted by inhibition of PRDX activity [46]. The inhibition of 2-Cys PRDXs or PRDX6 iPLA_2_ activity promoted a rise in ROS levels, identified by increased lipid peroxidation that did not impair sperm viability, but prevented phosphorylation of protein kinase A (PKA) substrates [47] and residues of tyrosine (a hallmark of sperm capacitation) [48,49,50], leading to the inhibition of human sperm capacitation [46]. 

The capacitated spermatozoon needs to undergo the acrosome reaction, an exocytotic event that releases hydrolytic enzymes, to penetrate the glycoprotein-made matrix called zona pellucida, which surrounds the oocyte, fuses with the oolemma, and fertilizes the oocyte [51]. We dissected this process to understand the role of PRDX6 iPLA_2_ activity in fertilization. The percentage of Prdx6^−/−^ spermatozoa to undergo acrosome reaction, to bind to the zona pellucida, and to fuse with the oolemma and fertilize the oocyte were lower than in the wild-type controls [15,27,52]. Moreover, wild-type spermatozoa treated with MJ33 during capacitation also had reduced percentages of these parameters compared to untreated controls. Altogether, these results suggest that the PRDX6 iPLA_2_ activity is essential for sperm fertility in humans and mice [15,27,46,52].

Different events occur at early, middle, and late stages of sperm capacitation. Under capacitating conditions, spermatozoa produce ROS, particularly superoxide anion, hydrogen peroxide, nitric oxide, and peroxynitrite, at concentrations that do not harm the cell but are necessary to trigger phosphorylation pathways [42,43]. We observed that MJ33 prevented the phosphorylation PKA substrates during human sperm capacitation [46]. This inhibitory effect was prevented when spermatozoa were capacitated with IBMX + dbcAMP, which increased the levels of cAMP bypassing the activation of PKA [47,49]. These findings indicate that PRDX6 iPLA_2_ activity participates in the early events of human sperm capacitation. Studies are ongoing to elucidate the role of PRDX6 during the early stages of sperm capacitation. 

The absence of PRDX6 or pharmacological inhibition of its iPLA_2_ activity by MJ33 promotes an increase in lipid peroxidation in the lung [53,54] and spermatozoa [15,27]. It is known that lipid peroxidation products, such as 4-HNE, can inactivate protein kinase C and extracellular signal-regulated kinase [55,56,57]. Because these kinases are needed for sperm capacitation [43,44,58], PRDX6 iPLA_2_ activity is essential to prevent the formation of products of lipid peroxidation that will produce inactive key enzymes necessary for sperm fertility.

## 5. Perspective and Future Research

There is a need for more research to elucidate whether there are close associations between PRDX6 and other antioxidant enzymes. Although we found that PRDX6 is the primary protector of the paternal genome [19], GPX5 and nuclear GPX4 are also important to protect DNA against oxidation [59,60]. However, contrary to Prdx6^−/−^ males [15] that show both in vivo and in vitro infertility [15,27], the nGpx4^−/−^ and Gpx5^−/−^ and the double GPX4/GPX5 knockout males are fertile [59,60,61]. DNA oxidation is higher in spermatozoa from the double GPX5/nGPX4 double-knockout male mice compared to WT controls, indicating that these enzymes are necessary to avoid DNA damage [61]. The level of other antioxidant enzymes, such as PRDX3, TRX, and GSTpi, are upregulated in the epididymis of the double-knockout males [61]. TRX and GSTpi participate in the reactivation of the peroxidase activity of 2-Cys PRDXs and PRDX6, respectively [6,13,19]. The absence of abnormal reproductive outcome (i.e., reduction of litter size, miscarriages, pups with developmental abnormalities) observed in adult males without nGPX4, GPX5, or both enzymes is an indication that other enzymes, such as PRDX6 and 2-Cys PRDXs, and their reactivation systems by TRX and GSTpi are acting to avoid significant oxidative damages, thus maintaining fertility in these modified mice. 

Contrary to females, male mice lacking SOD1 are fertile [62,63]. It is possible that in SOD^−/−^ males, there is an upregulation of PRDX6 and/or other antioxidant enzymes to lower the H_2_O_2_ levels produced by the increased levels of superoxide anion. Further studies are necessary to determine whether the absence of other antioxidant enzymes (i.e., GPX4, GPX5, SOD) along with PRDX6 have a greater negative impact on sperm quality. 

## 6. Conclusions

The limited protection of spermatozoa against oxidative stress resides in the architecture of this terminal cell. During spermatogenesis and epididymal maturation, part of the large cytoplasm of round and elongated spermatids is removed to generate the characteristic aerodynamic shape. Due to this removal, antioxidant enzymes, such as catalase and SOD, are removed from the forming spermatozoon. The only enzymes with antioxidant capacity are PRDXs, which can be easily thiol-oxidized and therefore inactive. Because ROS are needed for redox signaling associated with the acquisition of fertilizing ability, a delicate balance between ROS production and PRDXs must be established to assure the viability and functionality of the spermatozoon. Deficiencies in PRDX content and activity are plausible causes of male infertility. PRDX6 stands out of the family as the primary protector of the spermatozoon against oxidative stress to protect not only sperm function but the paternal genome, an essential cargo for the preservation of the species.
